# Does Physical Exercise Always Improve Bone Quality in Rats?

**DOI:** 10.3390/life10100217

**Published:** 2020-09-23

**Authors:** Hugues Portier, Delphine Benaitreau, Stéphane Pallu

**Affiliations:** 1 Laboratoire de Biologie Bioingénierie et Bioimagerie Ostéo-Articulaire (B3OA), Université Paris, UMR CNRS 7052, INSERM U1273, 10 Av de Verdun, 75010 Paris, France; stephane.pallu@univ-orleans.fr; 2 Collegium Science & Technique, 2 allée du château, Université d’Orléans. 45100 Orléans, France; dbenaitreau@yahoo.fr

**Keywords:** bone quality, physical exercise, rats, bone macro and microarchitecture, mechanical properties, osteocyte/osteoclast

## Abstract

For decades, the osteogenic effect from different physical activities on bone in rodents remained uncertain. This literature review presents for the first time the effects on five exercise models (treadmill running, wheel running, swimming, resistance training and vibration modes) in three different experimental rat groups (males, females, osteopenic) on bone quality. The bone parameters presented are bone mineral density, micro-architectural and mechanical properties, and osteoblast/osteocyte and osteoclast parameters. This review shows that physical activities have a positive effect (65% of the results) on bone status, but we clearly observed a difference amongst the different protocols. Even if treadmill running is the most used protocol, the resistance training constitutes the first exercise model in term of osteogenic effects (87% of the whole results obtained on this model). The less osteogenic model is the vibration mode procedure (31%). It clearly appears that the gender plays a role on the bone response to swimming and wheel running exercises. Besides, we did not observe negative results in the osteopenic population with impact training, wheel running and vibration activities. Moreover, about osteoblast/osteocyte parameters, we conclude that high impact and resistance exercise (such jumps and tower climbing) seems to increase bone formation more than running or aerobic exercise. Among the different protocols, literature has shown that the treadmill running procedure mainly induces osteogenic effects on the viability of the osteocyte lineage in both males and females or ovariectomized rats; running in voluntary wheels contributes to a negative effect on bone metabolism in older male models; whole-body vertical vibration is not an osteogenic exercise in female and ovariectomized rats; whereas swimming provides controversial results in female models. For osteoclast parameters only, running in a voluntary wheel for old males, the treadmill running program at high intensity in ovariectomized rats, and the swimming program in a specific ovariectomy condition have detrimental consequences.

## 1. Introduction

As the world’s population continues to age, the number of postmenopausal women, and potentially the incidence of osteoporosis, is increasing [1,2]. Approximately 50 million individuals met the WHO osteoporosis criteria in industrialized countries [3]. Other studies report that more than 200 million people suffer from this disease worldwide [1,4]. Close to a third of all postmenopausal women both in Europe and North America have osteoporosis. More than 40% of these osteoporotic women and more than 20% of men will experience bone fractures during their lifetime [5]. In the past, osteoporosis was not as common in men as in women, but since the last decade the risk of hip fractures in men has rapidly increased [6,7].

Prevention and treatment of osteoporosis and its related bone fractures is crucial for the quality of human life. Bone fractures are due to age, low bone mineral density (BMD), family history, premature menopause, previous fragility fractures and could also result from corticosteroid treatments [8,9,10,11]. Among other risk factors, hormonal deficiency, reduction in physical activities and important variations in body weight can also result in increased occurrence of hip fractures associated with decreased bone strength, which is a consequence of decreases in bone density and quality. Moreover, low BMD has been reported as the etiology of bone fracture frequency [12].

Improved understanding of the physiopathology of osteoporosis has led to the development of pharmacological treatments targeting BMD, bone turnover and fractures. Lifestyle, nutrition and physical exercise are other parameters pertinent to prevent osteoporosis [13,14,15,16].

There is evidence that physical exercise has unquestionable beneficial effects on health: for instance, regular aerobic fitness exercise reduces the risk of developing chronic diseases such as type 2 diabetes or colon cancer by 20–40% and the mortality associated with them by about 30% [17,18].

Bone tissue is a dynamic tissue that is modeled and reshaped according to the constraints applied to it (Law of Julius Wolff, 1892. [19]). In particular, the development of bone mass in the absence of exercise is estimated at only 30% to 50% of its potential [20]. As with muscle, exercise is therefore necessary for bone strengthening. Physical exercise causes mechanical stresses (impacts on the ground, shocks, traction at muscular insertions) that directly affect bone [21] and biochemical agents transported by blood (hormones, cytokines) favoring bone anabolism [22,23]. Exercise is also recognized to have a positive effect on the human skeleton and to contribute to the prevention and treatment of osteoporosis [24,25,26].

Following numerous experimental studies, it is now widely accepted that rats constitute a relevant model for exercise on bone [27]. Rat osteopenia reported after prolonged immobilization (hindlimb suspension), ovariectomy and aging is similar to human osteopenia regarding bone dynamics and anatomical features [27].

This outcome in experimental animals was achieved in the form of either forced or free exercise, using treadmill running, swimming or jumping. In other models, animals (usually rats) wore backpacks to increase weight or had to press levers of increasing resistance in order to get food rewards [28].

The effects of treadmill running on bone mass in female rats were reviewed by Iwamoto et al. in 2005 [29]. Since then, the interest in the effects of treadmill running on bone in healthy and in osteopenic rats has continued to grow. Moreover, other exercise models were developed including voluntary running in exercise wheels, swimming, resistance exercises (specifically jumping, drop, climbing) and passive exercise on vibrating platforms.

Because the results obtained in the different studies using the rat model are contradictory, there is no consensus regarding the effects of these various exercise programs on bone homeostasis/turnover.

Thus, it is important to study the effects of various types of physical exercise on bone in order to plan modern personalized preventive and therapeutic strategies for osteoporosis. Bone remodeling varies according to gender, age and peculiar underlying pathophysiological conditions. In turn, all these factors can variously interfere with the osteoanabolic effects of physical activity. Personalized physical exercise certainly represents, in association with drugs targeting immunological checkpoints, an important tool in the so-called precision therapy of osteoporosis.

**Figure 1:**
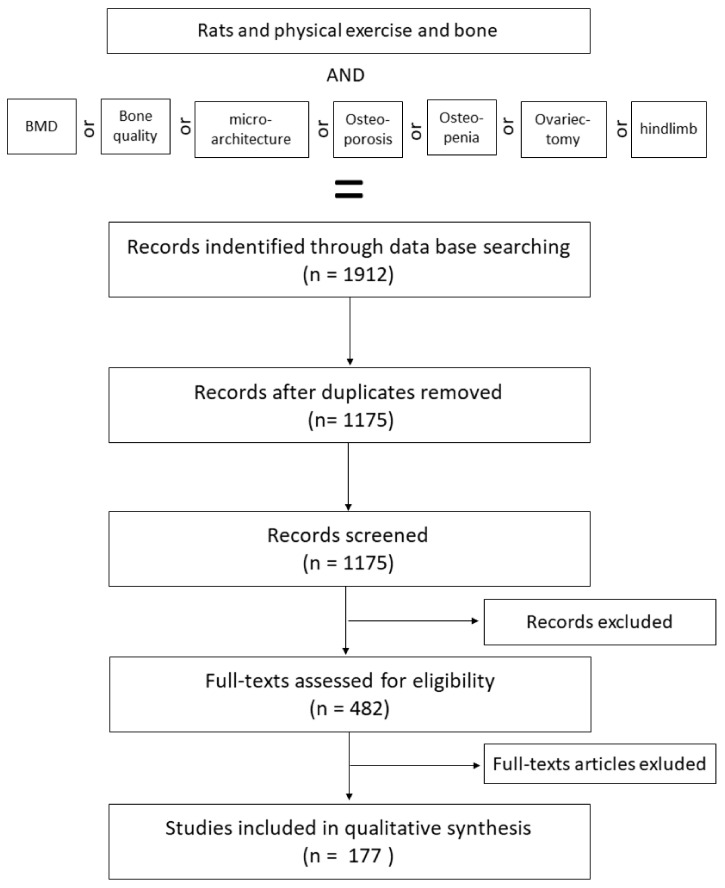
Flow of studies through the review.

In the present review, investigators searched PubMed (n = 1052), ScienceDirect (n = 131) and Google Scholar (n = 729) databases using the following strategy: (Rats) and (exercise/physical exercise) and (bone) and/or (bone mineral density/bone quality/microarchitecture) and/or (osteoporosis/osteopenia) and/or (ovariectomy/hindlimb) with English and French language restrictions. In total, 1912 publications were identified (Figure 1).

After these initial criteria, a second discrimination was made to separate the relevant studies from the others. First, a total of 737 duplicates (n = 358) and/or inappropriate languages (n = 109) and/or full text not available (n = 223) and other species (n = 47) were found and eliminated, leaving 1175 unique available citations with various exercise protocols and their effects on both male and female, well as healthy and osteopenic, rats were kept (n = 1175).

To finish the sorting, a total of 693 articles were excluded after scanning. The reasons for exclusion included studies with incomplete description of the species (n = 87), age of rats (n = 79), sex (n = 67), exercise protocol (n = 131) or bone parameters (n = 84), not a formal study (n = 28) and others (n = 217) were eliminated.

In addition, only the studies with physical exercise protocols that were controlled or for which the exercise load could be calculated were considered (n = 177).

## 2. Population and Bones Studied

### 2.1. Population Studied

All rat strains were included in this review. All selected studies on the effects of exercise on bone health were performed in healthy males and females or in osteopenic rats.

Management of osteoporosis and pertinent therapeutic modalities were investigated using animal models and clinical trials. Following numerous experimental studies, it is now widely accepted that rats constitute a relevant model for osteopenia [30]. Several procedures are used to induce osteopenia. In both males and females, gonadectomy was commonly used [27,31]. This operation induces an increase in bone resorption in detriment of bone formation. The ovariectomized rat model is an established and widely used animal model to study postmenopausal bone loss.

Another method for inducing osteopenia in rats is using prolonged immobilization by suspension. In 1979, to simulate bone changes induced by microgravity as experienced by astronauts or animals in space flights, Morey ER [32] developed a model in which the hind limbs of rodents were suspended by the tail. This model is a well-considered method to evaluate osteopenia caused by disuse/hypoactivity [33,34,35,36,37,38].

### 2.2. Bones Studied

The choice of the bone to be studied is important regarding physical activity. The tibia is the most distal bone and might be the one receiving the most important mechanical loadings, and thus shows the highest responses to physical exercise [39]. On the other hand, due to the more intense vibrations, it might also undergo lesions and develop fragilities and more fractures. The majority of the studies investigated effects on the femur, which is more proximal [40,41,42,43]. The vertebrae for their trabecular bone content are also frequently used, but do not undergo direct mechanical stimulation in physical exercise in quadrupeds [29]. Few studies also investigated bones that are not directly submitted to mechanical stress due to exercise, such as nasal bone [44].

## 3. Bone Parameters Studied

Bone strength is directly associated with bone quality and is dependent on a large variety of interconnected factors represented by the properties of bone tissue, which comprise the relative amount and biophysical properties of either the organic or the inorganic components. Bone geometry (bone size, cortical thickness, moment of inertia), microarchitecture (trabecular connectivity, trabecular shape, cortical porosity, tissue organization) and tissue properties (cellular density, osteocyte network integrity, mineralization degree, mineral crystallinity, hydration, degree and type of collagen cross-linking) all have an effect on bone quality [45].

### 3.1. Bone Mineral Density

Bone mineral density (BMD) is a parameter that reflects the mineralization of bone [45,46]. BMD measurements are usually performed using Dual-Energy X-ray Absorptiometry (DXA) [47]. These values can be measured for the whole body, a specific region of the skeleton (e.g., BMD of the lumbar region) or for a specific bone (e.g., femur). The most precise results are obtained when BMD is measured on a specific bone area (e.g., cortical part of the femur in the femoral neck) [48]. BMD is used to classify normopenic, osteopenic and osteoporotic patients using a standard age/BMD curve.

In addition, some other studies have measured BMD by quantitative computed tomography. This technique provides a volumetric (3-dimensional) BMD that has been shown to be a good indicator of bone quality and to correlate well with mechanical testing. Volumetric BMD differs from the areal (2-dimensional) BMD that is obtained using DXA [49,50,51,52].

### 3.2. Bone Micro-Architecture

The architecture aspect such as the thickness of the cortical part including the number and the thickness and distribution of the trabeculae are important parameters of bone strength. These bone parameters are assessed in rodents using X-ray microscanning [53] or histomorphometry [54]. For cortical parts, the most described parameter is the thickness (Ct.Th) [55]. The trabecular bone aspect evaluated is the bone volume/tissue volume (BV/TV), which reflects the percentage of trabeculae volume in the bone marrow, the trabecular thickness (Tb.Th) which is the average thickness of the trabeculae, and the trabecular number (Tb.N), which represents the number of trabeculae per volume unit. These parameters are associated with bone quality, whereas BMD is associated with bone quantity [45,56].

### 3.3. Bone Mechanical Properties

The mechanical parameters of bone were determined by ex vivo measurements of bone strength. Two types of tests are frequently used for these purposes: the three-point bending test, which measures the strength of long bones; and the compression test, which measures vertebrae and femoral neck resistance. Mathematical analyses of measurement of the bone deformation as a function of the applied strength provide values of the maximum strength before breaking, stiffness, and the Young Modulus; these parameters describe the mechanical properties of bone [57].

### 3.4. Osteocyte Lineage Biology

Osteocytes, the main cell constituent of mammalian bones, represent more than 95% of all bone cells (20,000 to 80,000 cells/mm^3^ in bone tissue). Over the last two decades, many publications have highlighted the role of this cell in bone homeostasis. The osteocyte is now considered as the initiator of the bone remodeling process [22]. Indeed, osteocytes, embedded in the mineral matrix, form an interconnected network of cells in bone structure which detect mechanical pressures and loadings.

Publications in the scientific literature highlight the positive impact of physical activity on bone quality in post-menopausal women [58,59]. The differentiation advance process in the osteoblastic/osteocyte lineage and its histologically linked parameters, such as mineral apposition and bone formation rates to different programs of physical activity (included absence of mechanical loading), are only documented in a few studies which used rodent experimental models.

Furthermore, mechanical stimulations of mouse osteocytes in culture in vitro attenuated apoptosis [60]. In contrast, reduction of mechanical loading in a tail-suspension murine model mimicking weightlessness increased the prevalence of osteocyte apoptosis, followed by osteoclast recruitment and associated bone resorption [60]. Osteocytes are cells playing a physiological role during both their lifetime, and through their apoptosis. Messages transmitted by osteocyte during apoptosis lead to the initiation of signals for remodeling. Several experimental exercise programs in rodents evidenced osteocyte viability as a biomarker of bone quality [61,62,63].

Consequently, knowledge of osteocyte biology parameters such as apoptosis, viability and differentiation biomarkers in rodent models allows for better understanding the effects of the different models of physical exercises.

### 3.5. Osteoclast Biology

Osteoclasts, derived from the macrophage lineage of hematopoietic stem cells, have the ability to resorb the calcified tissue. Osteoclast recruitment to the future resorption sites is mainly controlled by osteocytes and osteoblasts. Osteoblasts can express RANKL and Osteoprotegerin (OPG), the key factors that control osteoclastogenesis. RANKL binds to its receptor RANK on the surface of osteoclast progenitors and promotes osteoclast differentiation and enhances osteoclast activity [64]. In contrast, OPG prevents RANKL from binding to RANK, impeding osteoclast recruitment and differentiation. Different protocols of exercise can modulate bone resorption through the OPG/RANKL ratio. Besides, exercise can also regulate some serum osteoclastic markers such as the COOH-terminal collagen cross-links (CTX) [65,66] or tartrate-resistant acid phosphatase [67] and histomorphometric parameters of the resorption, such as the percent of active eroded surfaces, the number of osteoclast per trabecular bone surface or the trabecular osteoclasts surface [67,68]. Moderate exercise contributes to increasing the expression of OPG and decreasing the expression of RANKL and, as a consequence, to inhibit osteoclast differentiation and activity. Notomi et al. have previously demonstrated that resistance training increased the OPG expression in rats [69]. Treadmill and vibration stimulation training downregulated RANKL expression and upregulated OPG expression in bone cells in a rat model with glucocorticoid-induced osteoporosis [56]. Meanwhile, the knowledge of the effects of different exercise programs (running, walking, impacts, whole-body vibration, swimming…) on osteoclast biological parameters (viability, differentiation, activity) in rodent models is not very well documented in the literature.

## 4. Effects of Physical Exercises on Bone Health Status

### 4.1. Effects of Treadmill Running Exercises

#### 4.1.1. Treadmill Protocol Characteristics

In the scientific literature on bone in rodent models, most running treadmill protocols described running sessions at a constant intensity. Few studies investigated the effect of interval training, also called intermittent protocol [70,71].

These protocols, alternating high- or low-intensity sessions and rest periods, were developed as a special adaptation of interval training in humans to animals. Rest phases occur after a high session limit of the “desensitization” of osteocyte, allowing anabolic response [46,72,73]. Exposure to high sessions can induce a higher stimulation of mechanoreceptors in high-speed sessions [74], with a recovery of mechanoreceptors during the lower intensity sessions.

In most protocols reported in the scientific literature, the rats ran on a flat-bed treadmill, but some experimental studies used an inclined treadmill. Hamann et al. used both uphill and downhill slope and reported that this parameter might influence the results, thereby acting as a potent osteogenic stimulus [48]. In the literature, studies reported uphill inclination grades from 2° to 30° [49,75,76,77,78,79,80] or even downhill [81,82,83].

To generate a more intense exercise and/or load, the principle of weight bearing was applied on the vertebrae of animals. A backpack, representing up to 20% of body weight of the animal tested, was used during the whole running session [84,85,86,87,88,89].

To force rats to run, a horizontal grid could be positioned at the rear of each compartment of the treadmill, below which intermittent jets of compressed air were delivered.

Within 1–2 days of exposure subsequent to this exercise regime, the rats learned to run on the treadmill. For this reason, the air system was rarely used during exercise [75]. Variations of this system were developed, for example the frontal half of the treadmill was enclosed to respect the rat’s circadian system [90,91]. Alternatively, an electric grid at the end of each compartment was also used as a stimulus [91]. An electric stimulus (30 V, 0.5 A) can be manually turned on for less than 2 s when the tested animal stayed on the electric grid for longer than 10 s [90], but this method is reported to be stressful [91].

One of the main aspects of treadmill running studies is the very high variety of protocols that can be used. The parameters of speed, duration, frequency, additional weight and inclination give a large combination of possibilities.

It appears that speed (intensity) is an important parameter [92]. It can vary from a very low speed which will then reflect walking activity (13 m/min), to very intense running speeds (>25 m/min). Some teams have chosen to express and determine this speed as a percentage of the maximum aerobic speed that the animal can tolerate [72,93,94]. This technique can be very useful in determining optimal speed and generating a running protocol that is neither too intense nor too gentle. Another important aspect noted in the literature is the duration of either the session or the entire cycle. They lasted on average 60 min in a single bout for 8 to 12 weeks [65,66,90,95,96,97,98,99,100].

**Figure 2:**
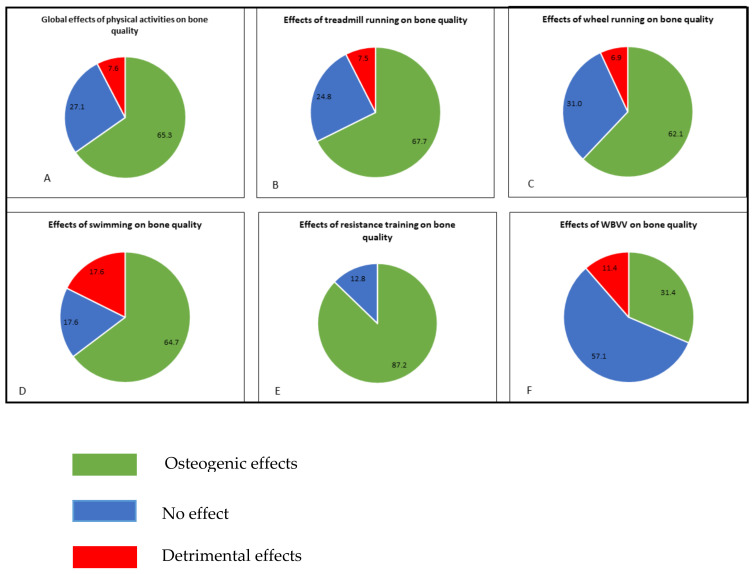
Global effects (**A**) and effects of each physical protocol (**B**): treadmill running, (**C**): wheel activities, (**D**): swimming, (**E**): resistance training, (**F**): VWBV (Vertical whole-body vibrations) on bone quality in the whole experimental procedures and reported results. Values are presented in percentage (%).

Treadmill is the most used protocol (n = 133 results in this review), with 67.7% positive results (Figure 2). The different effects of treadmill for the different bone parameters are summarized in Table S1.

#### 4.1.2. Specific Changes in Bone Mass, Structure and Strength

##### Healthy Rats

While many studies have examined the effects of treadmill running, the effects of exercise on BMD remain controversial and depend on gender, running protocols and bone status of the rats tested, without being able to determine a trend.

When four-week-old Wistar rats run for 10 weeks, (five days/week for 60 min) with a speed gradually increasing from 10 to 30 m/min, their BMD (measured by DXA) in the subtrochanteric, diaphyseal and the metaphyseal regions increased compared to their controls, but was similar at the proximal and distal ends of long bones of the two rat groups, suggesting that the stress distribution by running exercise differs along the skeletal sites [97].

In addition, several studies highlighted the positive effect of treadmill running on the femur and/or tibial, specifically increasing in the longitudinal and/or radial direction of the long bones after 4–5 weeks [48,101], 8 weeks [29,102], 10 weeks [78,97], 12 weeks [79,95] or 14 weeks or more [49,79,103,104,105].

Even if bone remodeling occurs as a long-time process, short protocols (four or five weeks) could provide an increased in BMD ([48,101] or in trabecular parameters [106]. Thus the question is asked [100] In addition, some researchers obtained positive effects in BMD with short sessions such as 5 to 10 min a day [49,79,103], or 30 min a day [44,48,102]. However, these results were made possible either by an additional load or by an up/downhill protocol to increase the mechanical loading.

In addition, another interesting parameter to study concerns protocols using a treadmill inclination. Hamann et al. described the effects of downhill running in rats [48]. The downhill protocol has a double interest. First, it has been shown that ground reaction forces (GRF) downhill were superior to those observed in level or uphill in humans [94,107,108,109]. Even if, to our knowledge, the GRF in downhill running has never been evaluated in rats, studies have been performed on other quadrupeds. Indeed, in dogs, GRF were evaluated in −15°, 0° and +15° treadmill inclination at 2 to 3 m/s. Despite the whole body, GRF was similar among conditions; the distribution of GRF differs between anterior and posterior limbs. Downhill running produced higher anterior GRF and uphill higher posterior GRF [110]. In the study by Hamann et al., only femur bone parameters were evaluated. In spite of the potential lower impacts on posterior limbs during downhill, the authors found higher BMD than on level [48]. Evaluating bone parameters on anterior limbs would be relevant. Second, downhill running is a natural form of eccentric exercise. In humans, it has been observed that the eccentric exercise performed on the ipsilateral leg produced +4% femur BMD gain in 18 weeks, whereas concentric exercise performed on the contralateral leg did not produce any effects on BMD [111]. That result is possibly related to the endocrine crosstalk between bone and muscle as proposed by Brotto & Johnson [112].

Hamann et al. suggested that there might be a critical strain threshold to stimulate bone formation [48]. Indeed, we could observe that no matter the gender or the age, short-term protocols and/or low-intensity protocols mainly had no effect. As an example, Maurel et al. observed no increase in whole-body BMD after exercise and proposed that this might be due to the moderate intensity of their exercise protocol [113]. Even if Hamann et al. classified their protocol as moderate exercise, they justified the fact that they separated the total daily duration in two bouts based on earlier studies that revealed an improved osteogenic response if a daily mechanical stimulus was partitioned into separate loading bouts [48,114]. Some authors have hypothesized that bone responsiveness to exercise should be bone-specific [48,97,115]. The tibia and femur are considered to be the most responsive bones, but this result can be biased as they are also the most frequently studied. Chang et al. chose to report lumbar BMD instead of distal femur or proximal tibia. They observed a higher variation in femoral BMD than in spine, and hypothesized that this might be due to the dimension of femoral bone (diameter) [116].

The last point concerning the characterization of the training load is the frequency of the sessions (per day and per week). A large majority of studies used one session per day, five times per week. Hagihara et al. have compared the effects of four, five, six or seven running sessions of 30 min of continuous running (15 m/min) on bone status. A gain in the BMD of the tibia was obtained after four, five and six days per week and an increase in the BMD for the femur was obtained after four or seven days per week [100].

In contrast to popular opinion, exercise does not always improve bone mass. Indeed, numerous studies suggested that BMD is not modified or even decreased by treadmill running exercise.

When rats (between five to eight weeks old) were exposed to treadmill running for 1 h/day, 2 to 27 weeks for a running speed from 10 to 30 m/min, the tibia and femur BMD were not affected [51,85,96,106,113,117,118,119,120,121,122,123,124].

For studies with the same duration and the same intensities, continuous treadmill running can lead to a decrease in BMD with values lower than those measured in the sedentary control groups [55,80,102,125,126,127].

Bone quality is also determined by micro-architecture, so some work has focused on the study of cancellous and bone. The first descriptions of bone microarchitecture in running female rats were made by Yeh et al. in 1993 [128].

Currently, it is very difficult to highlight a standard protocol that allows an improvement of the structural and architectural parameters of the bone. Indeed, for equivalent protocols in both male and female rats—mean age (five to eight weeks old), mean intensity (15 m/min, range 10 to 90 m/min), an intensity up to 30 m/min and a mean duration of 8 weeks or more—several studies highlighted the positive effect of treadmill running on the micro-architecture for the femur and/or tibial, specifically increases in the longitudinal and/or radial direction of the long bones, [68,77,97,105,106,121,125,128,129] coupled with an increase of cancellous parameters (i.e., BV/TV, Tb.Th or Tb.N) of the tibia or the distal femoral metaphysis [96,97,105,106,130].

In contrast, some other studies in the same conditions did not show any modification of trabecular parameters [48,79,85,120,131,132], and in the worst cases the training even induced a deterioration of these parameters for tibial proximal epiphyses and/or thoracic vertebrae [55,113,123].

The great majority of studies reported that treadmill running did not improve the bone mechanical properties in healthy exercised rats compared to sedentary rats. Maximal loading, ultimate strength, fracture load or Young’s modulus for the femur were not improve whatsoever for the time, intensity and duration of the protocols [48,49,71,118,133]. Only one study reported significant higher post-yield energy and ultimate energy in the femoral three-point bending test [90].

##### Osteopenic Models

Because osteoporosis is not a common pathology but still occurs in men, very few studies examined the effects of physical exercises on BMD in osteopenic male rats. The different protocols show that the stroke allows restoration of BMD after prolonged immobilization [134] or in an osteoporosis model by orchidectomy [104].

On the other hand, many studies have focused on the effects of treadmill running in osteoporotic female rats. However, as in healthy models, this type of exercise also does not lead to a systematic positive effect. Numerous studies for different protocols (i.e., duration and intensity) have shown a restored BMD compared to sedentary control rats but remained lower than in sham rats [65,85,135,136]. In contrast, some other studies failed to report a positive effect for running exercise [116,137].

In contrast to healthy animals, the effects of treadmill running exercise protocols in ostepenics/osteoporotic models seems to be more consensual. In most studies, the effects of exercise in osteopenic female rats counterbalanced BV/TV deterioration, but rarely increased gain in micro-architecture parameters beyond the starting values [44,138,139,140]. Furthermore, few studies failed to show a positive effect for such parameters [79,116].

Mechanical strains are very well studied in the literature because they correspond to a clinical reality. Numerous experimental studies describe very positive effects of exercise in mechanical properties in osteopenia models in the ovariectomized female but also in old rats [79]. From 7 to 17 weeks, the failure load of the femoral neck was higher than in control sedentary rats [56,65,66,76,133]. The stiffness or ultimate stress of the L5 vertebra was higher in running ovariectomized rats than in sedentary ovariectomized rats [67,92,98]. If treadmill running seems to be a good exercise to improve the bone mechanical properties on osteopenic/osteoporotic models, it remains that values were lower compared to control sham rats [65,66,92].

Very few studies have allowed this improvement for fast walking or running [103,120,137]. When rats were submitted to a hindlimb suspension, mechanical properties such as stiffness and ultimate load were not improved after running [98,141]. But in this case, the protocol to induce osteopenia was quite different and mechanical unloading induced a greater degree of bone loss than estrogen deprivation [142]. On the other hand, Iwamoto et al. demonstrated that there was no significant effect on lumbar bone mass after moderate exercise in OVX (ovariectomized) rats [136]. Sensitivity to exercise is not only bone-specific but also gender-specific, showing that male rodents’ responsiveness to moderate running exercise is more pronounced than for females [143].

#### 4.1.3. Specific Changes in Osteoblast/Osteocyte Lineage Biology

##### Healthy Rats

When the cellular responses were observed in male experiment models, literature has shown that treadmill running procedure (either moderate continuous or interval training running) mainly induced positive effects on the number or the viability of the osteoblastic/osteocytic lineage [61,62,93,143,144].

Deleterious effects on osteoblast lineage were only observed in male rats and for intensive treadmill running at 80% of maximal O_2_ consumption [55,117]. A majority of publications have highlighted positive bone osteogenic effects of the moderate continuous running treadmill procedure on osteocyte in female rats [65,66,145,146,147], whatever the age, at the end of the experiment.

Furthermore, the literature did not show negative results on histomorphometric parameters and the viability of the osteoblast/osteocyte lineage in female rats exercise effects on bone health status remained without effects [125,143].

##### Osteopenic Models

In osteopenic models, as previously shown for female rats, whatever the running mode (moderate continuous or interval training), the literature did not show negative results on osteogenic histomorphometric parameters and cell viability from the osteoblast/osteocyte lineage [59,66,139,140,145,148,149].

#### 4.1.4. Specific Changes in Osteoclast Biology

For osteoclast parameters (osteoclast number, osteoclast surface), treadmill running procedures provoke positive effects by decreasing resorption parameters and is observed both in male and female healthy models [68,125,150].

Moreover, treadmill running procedures induced osteogenic effects by decreasing osteoclast parameters in the different OVX models, except for the rats submitted to the highest speed (30 m/min) with no effects [66,128,140]. All these results and observations highlight the fact that treadmill running has to be considered as a preventive treatment more than a complete healing protocol. Too high intensity running might be deleterious for bone quality, inducing micro-cracks. Too low intensity is not sufficient to show a good mechano-stimulation of bone. Indeed, the choice of the protocol length and modalities is essential in conducting exercise studies on bone in rats.

### 4.2. Voluntary Wheel Running Protocols

#### 4.2.1. Protocol Characteristics

This method presents an absence of stress for the animals which could run when they want and keep their own environment. The nocturnal activity [151] and natural intermittent activity of the rats can be maintained [30].

Newhall et al. reported two advantages of voluntary exercise by showing that rats freely ran further (3–18 kms/day) and that the natural activity was less stressful than forced activity [135]. However, if the rats have free access to the running wheel all day long during the whole duration of the study, the effective time spent running and the distance achieved are highly variable. Animals are usually running exclusively during the light-off periods [151] and distance of run is increasing over the time of the experiment, reaching more than 300 min/day in male rats [151]. Some rats have been reported to run 5 kms/day [151], 10 kms/day [30], and 14 kms/day [152]. With aging (around 5 months), the daily distance decreases [30,123,152]. Female rats seemed to run higher distances than male rats [153] and chemical gonadectomy using goserelin acetate reduced wheel running distance significantly in both genders [153]. It was also observed that ovariectomized females Wistar displayed an eight times lower movement activity than sham rats [154].

The natural intermittence activity is less stressful than forced activity [30]. Meanwhile, the use of voluntary running in experimentation presents some other limits. First, voluntary exercised rats weighed less than age-matched sedentary controls. To limit the influence of this parameter, Newhall et al. proposed to match the rats to a similar weighted control rather than to a similar aged control, considering that the weight influences the bone results more than the age [135]. A second problem in wheel running protocols could be the difference observed between rats in running distances. Because the access to the wheel is free and not forced, it is very difficult to have a standardization of the running time, the distance, and the intensity of the exercise.

All results of wheel activities are grouped in Table S1. Overall, 62.1% of the results reported obtained a positive effect on the bone quality (Figure 2). The different effects of wheel running for the different bone parameters are summarized in the Table S1.

#### 4.2.2. Specific Changes in Bone Mass, Structure and Strength

##### Healthy Rats

In wheel running protocols, the distance traveled by animals was extremely variable (3 to 18 kms) [135]. No positive correlation was found between the distance ran and BMD, cross-sectional area, or polar moment of inertia in male Sprague Dawley rats [30,135,151,153,155].

Almost all the healthy running rats where described to have higher BMD than matched controls at various sites (tibia and femur) [115,135,155,156,157]. Only one study by Sipos et al., showed no statistical difference neither in tibia nor in femur BMDs between either controls, treadmill or voluntary wheel trained rats after an 18-month experimentation on male Sprague Dawley rats aged 23 months at the end of the study [123].

BV/TV, Tb.N and Tb.Th, trabecular, cortical thickness and mechanical properties were increased in male and female rats after six weeks to four months of physical activity [30,151,155] when compared to sedentary control groups. All the bones of the rat’s paw and all sites could be concerned by the improvement [119,155,157]. It should be noted that this type of exercise did not seem to have a negative effect even if few studies showed no statistical difference neither in tibia nor in femur [123,155].

##### Osteopenic Models

In contrast with treadmill protocols, the wheel running seems less effective in correcting the deleterious effects of gonadectomy (i.e., treatment with goserelin acetate, ovariectomy), with a non-significant protection in bone loss whatever the sex, the sites and the parameters studied [52,153,154].

Wheel running seems to induce important increases in lower limb BMD in healthy rats but seems to have little or no effect in restoring the bone mineral content in osteopenic rats. Moreover, one study described a negative effect of a such voluntary exercise on bone quality in ovariectomized rats [158]. Indeed, histomorphometric analysis of femur mid-diaphysis cortical bone revealed that this exercise provoked a significantly smaller osteocyte number in the OVX exercised group and could contribute to prevent osteocyte death in cortical bone, and also slow down the osteocyte differentiation aging in female experimental condition. Meanwhile, running in voluntary wheels has contributed to a negative effect on bone metabolism in old male experimental models [123].

Very few works have described effects of voluntary wheel running (or walking) on osteoclast activity and the results are controversial [123,159].

#### 4.2.3. Specific Changes in Osteoblast/Osteocyte Lineage Biology

##### Healthy Rats

Very few publications have described the effects of the voluntary wheel running modality on osteoblast/osteocyte lineage biology in heathy male and female rats [123,159].

For voluntary wheel running, Sipos et al. submitted 23-month old male Sprague Dawley rats to three different housing conditions from the age of five months: (i) rats housed individually with voluntary running wheels attached to cages, (ii) dieting rats housed individually fed to pair weight with running rats, (iii) walking rats exercised mildly by use of a treadmill (800 m/day, five days a week), (iv) rats housed as four in a cage and fed ad libitum [123].

Between these four groups, osteoblasts of running rats were not only decreased in number but displayed a lower activity as indicated by decreased serum osteocalcin levels. Running in voluntary wheels has a negative effect on bone metabolism in these old male rats [123].

In Fonseca et al., 2011, female Wistar rats (five months old at the beginning) were either housed in cages with or without a voluntary running wheel or sacrificed at age 14 months. Histomorphometric analysis of femur mid-diaphysis cortical bone revealed a significantly higher osteocyte number and lower empty lacunae number in the exercise group compared to their sedentary counterpart [158].

##### Osteopenic Models

Solely two studies described a positive effect of a voluntary exercise running on bone strength in ovariectomized (OVX) rats [158,160].

Indeed, histomorphometric analysis of femur mid-diaphysis cortical bone revealed that the voluntary wheel running procedure provoked a significantly higher osteocyte number and lower empty osteocyte lacunae number according to the OVX group [158].

Goulet et al. 2011, compared a rat model of high-capacity and low-capacity runners (HCRs and LCRs, respectively: Koch-Britton rat model of high-capacity and low-capacity runners). This study evaluated the effects of aerobic capacity on measures of bone mass and strength as well as osteoblast activity following ovariectomy (OVX). They hypothesized that intrinsically high aerobic capacity would increase osteoblast response. At 4 weeks post-ovariectomy, HCRs demonstrated a more robust osteoblast response [160].

HCR OVX rats demonstrated a more robust upregulation of osteoblast activity compared to LCR OVX rats. Proximal tibial metaphyseal mineral apposition rate (MAR) was also significantly upregulated to a greater extent in HCRs relative to LCRs. Markers of bone formation were upregulated to a greater extent in HCRs than LCRs, suggesting a role for aerobic capacity in governing osteoblast activity [160].

To conclude on osteocyte parameter, we have noticed that whatever the experimental model (OVX, female), voluntary wheel running could contribute to prevent osteocyte death in cortical bone, and also slow down the osteocyte differentiation aging in female experimental condition. Meanwhile, running in voluntary wheels has contributed to a negative effect on bone metabolism in the old male experimental model [123].

#### 4.2.4. Specific Changes in Osteoclast Biology

##### Healthy Rats

Very few works have described the effects of voluntary wheel running (or walking) on the osteoclast activity [123,159]. In male rats (five months old Sprague Dawley), Sipos et al. compared four experimental groups in 18 months: (i) a running wheel group, (ii) dieting rats housed individually, (iii) walking rats on treadmill, (iv) four rats in one cage. Sipos et al. demonstrated that the running group exhibits the highest serum terminal collagen cross-links (CTX) levels. This was in the same experimental group where osteoblasts were not only decreased in number, but displayed a lower activity as indicated by decreased serum osteocalcin levels. Running in these voluntary wheels has negative effects on bone metabolism in these old males [123].

Lertsinthai et al. investigated whether a voluntary wheel running program can rescue a four week –CAS (Chronic Aversive Stimuli) exposure on trabecular bone loss in eight-weeks-old OVX rats. At the sacrifice, they have shown that the active erosion surface (a.ES/BS) was lower in the OVX + CAS + running condition vs. the OVX + CAS group, which shows that wheel running had positive effects on osteoclast parameters in these 13-week-old OVX rats submitted to chronic aversive stimuli [159].

### 4.3. Effects of Swimming Exercises

#### 4.3.1. Swimming Protocol Characteristics

In human studies, while weight bearing physical activities have been shown to improve BMD, several studies reported that non-weight bearing activities, such as swimming, do not seem to improve BMD [161,162].

In animal studies, the swimming protocol usually consists of swimming in a water tank where the animals cannot touch the bottom or hang on the sides. The body weight bearing action of the muscles and long bones of the extremities is reduced significantly. Under these conditions, the bone modeling effect of swimming can be attributed only to the torque and pulling action on the bones by the muscle contractions. The animals swam one hour, five days/week, in nearly every study reviewed here, except one study where they swam only 30 min per day [134]. Some experiments include a variation of exercise intensity using weights attached to the tail during the swimming, from 0% to 5% of the animal’s body weight [81,163,164,165,166,167]. Duration of the swimming protocols is highly variable upon different studies (three weeks up to 20 weeks).

This exercise protocol is characterized with the highest percentage of the negative results amongst all the protocols used (17.6%, Figure 2). Whole swimming results are grouped in Table S1.

#### 4.3.2. Specific Changes in Bone Mass, Structure and Strength

##### Healthy Rats

The effects of a non-weight bearing exercise seem to be not clear concerning BMD and bone morphology, and the bone mechanical parameters are inconsistently modified.

For male and female rats, whatever the age, the literature shows contradictory results for swimming protocols. Three or 10 weeks have permitted to increase vertebrae or humerus BMD in both sexes [81,164], when Huang et al. in 2003 found no difference after 8 weeks of a swimming program [167] and while other studies showed negative effects, which was no longer significant when the BMD was adjusted to the rat body weight [166].

For an eight-week protocol period, structural bone parameters for tibia, femur, humerus or vertebrae characterized by BV/TV, Tb.N and Tb.Sp (primary spongiosa) and Tb.Th (secondary spongiosa) could either not be modified [163] or could be altered in both males and females [166,168]. On the other hand, after eight weeks of training, swimming improved ultimate load, impaired maximum load, and did not modify stiffness [165]. When the duration of the protocol was extended to 20 weeks, there was a gain of bone strength due to increased periosteal apposition and modified bone tissue distribution [163].

An interesting hypothesis was proposed by Huang et al. in a comparative study where swimming rats and running rats were analyzed. In this study, an increase of the water content of swimming rats’ bone was observed [165]. This effect on water bone content was previously observed in two other studies in female rats [164,169]. Even if the exact role of water content in bone tissue is not fully understood, its contribution to stabilizing collagen [96,170] might exert an influence on the mechanical properties of bone. Previously, in vitro studies have shown that hydration level of bone greatly influences its biomechanical properties [171,172].

They hypothesized that water bone binding might stabilize the collagen and thus modify the mechanical properties of the bone. The effects of this non-weight bearing exercise on bone strength might thus not be linked to the macroscopical structure of the bone, but to its microscopical structure and chemical content [165].

##### Osteopenic Models

After 14 weeks in ovariectomized rats, swimming had a positive effect on bone mineral properties indicated by greater BMD [134], mechanical properties and histomorphometric indexes compared with the control group. The greater cortical area and smaller minimum cortical width observed in the swimming compared with the control group are consistent with the greater BMD and better biomechanical properties observed as a result of swim exercise [173].

#### 4.3.3. Specific Changes in Osteoblast/Osteocyte Lineage Biology

##### Healthy Rats

Very few studies have explored the effect of swimming on the cell viability and the differentiation potentiality of the osteoblast/osteocyte lineage [168].

Bourrin et al. built a program of five weeks of swimming (2–6 h per day including two rest periods, five days a week) for 10-week-old swimming females. Their histomorphometric parameters showed that osteoid surface and eroded surface were lower in all evaluated locations of swimming rats, suggesting a decreased bone turnover [168].

#### 4.3.4. Specific Changes in Osteoclast Biology

Falcai et al. compared the swimming and hindlimb unloading condition with the hindlimb unloading condition alone. They measured that the swimming and hindlimb unloading condition did not present a significant difference for the osteoclast number, the eroded surface and the osteoclastic surface according to the hindlimb unloading context [174].

Furthermore, for the ovariectomized female rats, as previously shown, Lertsinthai et al. investigated whether a four-week CAS exposure aggravated trabecular bone loss in ovariectomized rats [159]. This CAS program included a swimming part with a swimming frequency at 1 h/day, five days/week for four weeks. They demonstrated that the osteoclast surface (Oc.S/BS) in the proximal tibial metaphysis was significantly higher in the OVX+CAS group compared to the OVX group. Moreover, the active erosion surface (a.ES/BS) was significantly higher in the OVX+CAS group compared to the OVX group. Consequently, this CAS program, which included a swimming section, had bone detrimental consequences on the osteoclast parameters on 13-week-old ovariectomized Wistar rats [159].

### 4.4. Effects of Resistance Exercises

Because aerobic (i.e., running or swimming) exercises are not always osteogenic models, other exercise methods have been developed to mimic impact activities in humans (i.e., collective sports), considered to be a better osteogenic model [120,175,176]. In rats, this exercise model presented the highest percentage of osteogenic effect (87%, Figure 2). Results for resistance training parameters are grouped in Table S1.

#### 4.4.1. Resistance Protocol Characteristics

Among resistance training, several types of protocols are proposed. Some can be models of jumping [177,178], free-fall impacts [50], isometric strength training [179] or climbing [180].

For jumping exercise, each rat jumped and grasped the top of a wooden box (around 40 cm in height) with their forelimbs and pulled themselves over the edge; the rats were then returned to the bottom of the box by hand [177,178,181,182]. Most of the time, the initial jump height was around 25 cm, which was gradually increased to 40 cm by the end of the first week [33,34]. Moreover, at the beginning, the rats were conditioned to jump with the help of an electrical stimulation.

This form of protocol was usually performed five times a week and rats jumped 10 times/day. Various interval durations were employed: 3 s [35,182] to 30 s [182]. In some studies, additional weights can be used with Velcro vests until more than 400 g [183]. Some other studies have used a number of jumps per day higher than 10 (up to 100) [35,181,182,183,184,185,186] or a double jumping session, in the morning then in the afternoon [182,185,186].

For the free-fall impacts, the rat was lifted horizontally until its feet reached the specified height from 30 to 60 cm [50,187,188], then the rat was released with a specific frequency between the drops [50,187] so that it landed on all feet approximately simultaneously onto a bare floor. The protocol was visually monitored to verify the accuracy of the landings [50]. Interval durations used were 10–11 s [50,187] based on a study using a four-point bending model [46]. The number of impacts per session varied from 10 [50,187,188] to 30 [188].

For climbing, rats were housed in a metal cage with a wire-mesh tower, with two water bottles set at the top (18). At the beginning, the bottles were set at a height of 20 cm. The set point of the drinking bottles was gradually elevated to 200 cm. The daily distances and time periods of climbing activity were obtained from the monitoring records [180,189].

#### 4.4.2. Specific Changes in Bone Mass, Structure and Strength

High-impact exercises (i.e., jumps) are more beneficial for bone than moderate impact exercises (i.e., running) [33]. Indeed, the benefit of impact exercise is thought to result from the dynamic nature and high strain rate, as well as the magnitude of mechanical stress imposed by high-impact exercise on bone. In 2014, and for the first time, the comparison of active upward jumps (40 cm) vs. passive drop jumps (40 and 60 cm) was proposed by Ju et al. The training consisted of 10 jumps per day, with 11 s interval, five days/week for eight weeks. Contrary to their hypothesis, the authors found more osteogenic effects on the distal femoral metaphysis using an active upward jump compared to passive drop jumps despite higher ground reaction forces for drop jumps. The authors suggested that concentric muscle action of an upward jump would lead to a higher mechanical stress transmitted to the bone than the eccentric muscle action of a drop jump [190]. In our opinion, it must be noticed that passive drop jumps exert more bone effects on forelimbs than hindlimbs and might explain these results [50].

In both male and female rats whatever the age for jumps performed between 30 to 60 cm, 10 to 50 times per day, three to five days per week for around eight weeks, BMD was increased compared sedentary control [50,177,183,185,187,191,192]. Moreover, for the same protocol, the effects could be different according to the sites observed [186,193], with occasional but rarely no effect on BMD (i.e., femur in sham group) [193]. It is noted that the rest time between jumps does not seem to have any effect on the results [50,182].

For the microarchitecture, the results were just as interesting, with some studies showing an increase in BV.TV, Tb.Sp, Tb.N evaluated by micro-computed tomography or histomorphometry ([183,190] when very few studies failed to have a gain for the trabecular bone [177].

The strength of bone (i.e., mechanical properties) was improved for numerous studies, whether it be the maximum load, the stiffness, the ultimate breaking or Young’s modulus [50,177,181,182,186,187,194]. Despite this, some studies have not shown positive effects on the mechanical properties [184,188].

##### Osteopenic Model

All the studies have shown an improvement of BMD or microarchitecture parameters or mechanical parameters after a resistance training [33,34,35,174,178,185,190,191,193,195,196]. However, some studies revealed a disparity in efficiency between bones (tibia versus femur) [196].

Moreover, for most of the studies the gain did not allow for reaching the values of the sham groups.

#### 4.4.3. Specific Changes in Osteoblast/Osteocyte Lineage Biology

##### Healthy Rats

There is no publication on the effect of resistance exercise on the modulation of the osteocyte differentiation lineage in rats, meanwhile Notomi’s team has studied the effect of such protocols on histomorphometric bone formation parameters.

Notomi et al. determined the effects of resistance versus aerobic exercise on bone turnover. In their study, male Sprague Dawley (four weeks of age) were assigned to one of three experimental groups: sedentary, running or jumping. In their jumping group, the trunk was kept upright during electrically stimulated jumping for 1 h every day. The running rats ran at speeds of 24 m/min for 1 h every day. The jumping group was trained at the same time of day on each occasion [197]. The rats wore a leather jacket connected to 35-cm wooden bar, the other end of which was attached to a fulcrum that was secured to a table. The duration of the jumping was equalized to that of running.

After four weeks, the jumping rats exhibited an increase of the trabecular mid-femoral periosteal bone formation rate per bone surface (BFR/BS). Furthermore, only the jumping rats showed a decrease in the BFR/BS at the endocortical surface. In conclusion, their results suggested that resistance exercise accelerated the cortical drift and increased the bone mass by stimulating bone formation more efficiently than running.

Notomi et al. determined the effects of the tower climbing exercise on the local bone turnover with male Sprague Dawley rats, 10 weeks of age, assigned to five groups: a baseline control and two groups of sedentary and exercise rats. Rats voluntarily climbed the 200-cm tower to drink water from the bottle set at the top of it. After four weeks, the trabecular bone formation rate (BFR/bone surface (BS)) of both the lumbar vertebra and tibia increased [180]. After eight weeks, at the femoral midshaft, MS/BS, MAR, and BFR/BS parameters were measured in the periosteal and endocortical envelopes. Authors have demonstrated that the periosteal MS/BS, MAR, and BFR/BS increased, although the endosteal MS/BS, MAR, and BFR/BS decreased.

The beneficial effects of mechanical loading on bone are not fully understood. Turner et al. stated that osteocytes, osteoblasts and bone-lining cells are influenced by strain-induced alterations in canalicular fluid flow. Then, through different mechanisms, e.g., growth factors, prostaglandins, or other mediators, osteoblasts are locally influenced to increase the production of bone matrix [84].

In male experimental models, SRT during unloading exhibited much lower prevalence of sclerostin-positive osteocytes according to the hindlimb unloading condition [198] and contributed to slowing down the osteocyte differentiation aging.

##### Osteopenic Models

In male experimental models, hindlimb unloading contributed to a lower MAR and bone formation rate according to ambulatory rat controls [84,150,199], and also to a significantly greater percentage of apoptotic cancellous osteocytes [199]. Simulated resistance training (SRT) programs during hindlimb unloading exhibited a lower prevalence of sclerostin-positive osteocytes according to hindlimb unloading condition [198] and contributed to slowing down the osteocyte differentiation aging.

Falcai et al. developed an original program of comparison of different protocols of exercise applied on a hind limb-suspended model in female Wistar rats [174]. Their objective was to compare the effects of swimming, jumping and vibration therapies on the prevention of bone loss. Their results have shown that whatever the training exercise program, the trained rats showed a significant increase in bone formation according to the hind limb-suspended condition alone, with no differences between exercise groups.

To determine the effects of a tower climbing exercise on mass, strength and local turnover of bone, nine-week-old Sprague Dawley male rats were assigned to seven groups: a baseline control and three groups of sham-operated sedentary, orchidectomized (ORX)-sedentary and ORX-exercise rats. In the exercise group, rats voluntarily climbed a 200-cm tower to drink water from a bottle set at the top [189].

Their results have shown that at the fourth week, the periosteal bone formation rate (BFR) at the mid-femur was maintained in ORX-exercise rats, whereas these parameters were reduced in ORX-sedentary rats. At the eighth week, the periosteal mineral apposition rate and BFR in ORX-exercise rats was significantly higher than the other groups.

Moreover, they have also demonstrated that at the eighth week, in ORX-exercise rats the trabecular mineralizing surface, BFR and bone volume of the lumbar vertebrae were maintained at the same levels as those in the sham-sedentary group [189].

The same team built an experimental study on ovariectomized Sprague Dawley rats. In this work, they achieved two consecutive experiments. In their first experiment, Sprague Dawley rats, 12 months of age, were assigned to four groups: a baseline control, sham-operated sedentary, OVX-sedentary and OVX-exercise rats. As previously shown for male rats [189], OVX-exercise rats voluntarily climbed a 200-cm tower to drink water from a bottle set at the top, and the exercise began three days after the ovariectomy. After three months, bone histomorphometry was achieved at the lumbar vertebral body (L3 and L4) and at the femoral mid-shaft. They measured the L4 vertebrae, the bone formation parameters, Mineral Apposition Rate (MAR, μm/day), the mineralizing surface per bone surface (MS/BS; %) and the surface referent bone formation rate (BFR/BS; μm^3^/μm^2^/day).

Their results about dynamic parameters at the mid-femoral diaphysis and the periosteal surface have shown that in the OVX-exercise group, all the parameters were significantly lower than those in the OVX-sedentary group. Furthermore, at the endosteal surface, the MS/BS, MAR and BFR/BS values in the OVX-exercise group were lower than in the OVX-sedentary group.

About the bone formation parameters studied at the lumbar vertebrae, authors showed that all the values in the OVX-exercise group were significantly lower than those in the OVX-sedentary group. Authors concluded at the end of this first part that such an exercise prevented OVX-induced cortical and trabecular bone loss by depressing turnover elevation.

In a second experiment, authors measured the recovery effect of the exercise element, which is rarely studied in the literature. In this part, Sprague Dawley rats, 12 months of age, were assigned to six groups: a baseline control, two groups of sham-operated sedentary and OVX-sedentary, and OVX-exercise rats. The climbing exercise started three months after the OVX operation. After six months of the experiment, in the OVX-exercise group all values of the dynamic parameters of the periosteal at the mid-femur surface were significantly higher than those in the OVX-sedentary group. At the endosteal surface, the same parameters were significantly lower.

About bone formation parameters acquired at the lumbar vertebrae, MS/BS, MAR and BFR/BS parameters were significantly higher than those in the OVX-sedentary group. These results confirmed that the climbing exercise prevented negative changes in cortical bone structure.

#### 4.4.4. Specific Changes in Osteoclast Biology

##### Healthy Rats

In their study where they compared jumping and running, Notomi et al. determined the effects of both modalities on osteoclast biology. After four weeks, they showed that at the lumbar vertebrae L3, the parameter Oc.S/BS was lower than the same parameter in both the sedentary and running groups [197].

Notomi et al. also determined the effects of the tower climbing exercise on the local osteoclast biology in male Sprague Dawley rats, 10 weeks of age, previously assigned to five groups: baseline control and two groups of sedentary and exercise rats. After eight weeks, Oc.S/BS values were significantly lower in the exercise group compared to the sedentary groups [180].

##### Osteopenic Models

About jumping, Falcai et al. noticed that the hindlimb suspension and jumping condition had a lower osteoclast number than for the hindlimb suspension interrupted by a regular weight-bearing condition. The eroded surface and the osteoclastic surface were lower in the hindlimb suspension and jumping condition compared to the hindlimb suspension interrupted by regular weight-bearing condition; this result confirmed an osteogenic effect of the jumping context on the osteoclast parameters according to the hindlimb suspension [174].

The whole of these works has demonstrated that jumping/SRT programs have osteogenic effects on the osteoclast parameters, whatever the animal sex [36,198].

Notomi et al. determined the effects of a tower climbing exercise on the osteoclast biology of bone with nine-week-old Sprague Dawley male rats assigned to seven groups: a baseline control, three groups of sham-operated sedentary, orchidectomized (ORX)-sedentary and ORX-exercise rats [197]. They demonstrated that at the eighth week, in ORX-exercise rats the osteoclast surface decreased compared with the ORX-sedentary group.

Notomi et al. also built an experimental study on ovariectomized Sprague Dawley rats [200]. In this work, they achieved two consecutive experiments. In their first experiment, Sprague Dawley rats, 12 months of age, were assigned to four groups baseline control, sham-operated sedentary, OVX-sedentary and OVX-exercise rats (tower climbing). The exercise began three days after the ovariectomy. After three months the bone resorption parameters studied at the lumbar vertebrae showed that the values in the OVX-exercise group were significantly lower than those in the OVX-sedentary group.

In a second experiment, the climbing exercise started three months after the OVX operation. After six months of experiment, in the OVX-exercise group, at the lumbar vertebrae Oc.S/BS was significantly lower than the same parameter in OVX-sedentary group.

In a female model, histomorphometric parameters of bone formation and serum levels of bone markers show that jumping provoked a significant increase in bone formation and serum levels of bone markers according to the hindlimb unloading condition [174]. They noticed that the hindlimb suspension and jumping condition had a lower osteoclast number than for hindlimb suspension interrupted by a regular weight-bearing condition. The eroded surface and the osteoclastic surface were lower in the hindlimb suspension and jumping condition compared to the hindlimb suspension interrupted by regular weight-bearing condition; this result confirms an osteogenic effect of the jumping context on the osteoclast parameters according to the hindlimb suspension.

The whole of these works have demonstrated that jumping/SRT programs have positive effects on the osteocyte and osteoclast parameters, whatever the animal sex [174,198].

About the tower climbing procedures, the main results of the effects of this exercise on osteoblast/osteocyte biology and osteoclast parameters have been studied by Notomi’s team [180,189,197,200]. The whole of these works has shown that such an exercise modality can have beneficial effect on cortical bone at a cellular level (osteoclast, osteoblast) by accelerating the cortical drift or by preventing bone loss by depressing turnover elevation (in osteopenia models). It is important to note that resistance training never induced adverse effects for all parameters.

### 4.5. Whole-Body Vibrations Exercise

#### 4.5.1. Whole-Body Vibrations Protocol Characteristics

Since the 2000s, a new type of exercise has appeared in the fitness industry: exercise on a vibrating platform where vibrations are a mechanical stimulus characterized by an oscillatory motion. The principles of whole-body vibration (WBV) lie in the law of motion as stated by Newton: mainly, that the force (F) of an object is equal to the mass (M) multiplied by its acceleration (A) (F = M × A). Whole-body vibration machines utilize acceleration by keeping the body weight constant [201].

Intensity may be determined by the amplitude, frequency and magnitude of the oscillations. The extent of the oscillatory motion determines the amplitude (peak-to-peak displacement, in mm) of the vibration, the repetition rate of the cycles of oscillation denotes the frequency of the vibration (measured in Hz), and the acceleration indicates the magnitude of the vibration. Whole-body vibration training (WBVT) has been used as a supplement to conventional exercise training, such as resistance exercise training to improve skeletal muscle strength, specifically in the rehabilitation field [202]. For these protocols, rats were placed in a single or collective cage on a vibrating platform. Recently, studies have focused on the application of high loading frequencies (>10 Hz), commonly referred to as vibrations [202]. Indeed, the high frequencies used are ranged around 12–30 Hz [203] and 45 Hz [204] to 150 Hz [205]. Acceleration ranged between around 0.3 g [206] to 4.9 g [207].

Amongst the five exercise protocols, this kind of mechanical strain seems to be the less efficient (positive effects: 31.4%, Figure 2). WBVV results are grouped in Table S1.

#### 4.5.2. Specific Changes in Bone Mass, Structure and Strength

There are very few works about the WBVV in healthy models. Most of studies focus on ovariectomized female rats.

The majority of the studies do not show any effect in bone mass, structure and strength, whatever the training period: 14 days, 35 days or 12 weeks intensity (one or twice stimulations per day), with an intensity ranging between 8 to 150 Hz and amplitude around 0.5 mm and 0.2 to 3.9 g [207,208,209,210,211,212]. In contrast, when the vibrations were applied for 12 weeks (5 d/w, 30 min/d) to seven-month-old female rats, whole-body BMD, cancellous bone volume and architecture in the first lumbar vertebra increased [213].

Moreover, Xie et al. showed that a prolonged WBVV treatment (16 weeks) caused significant reduction in the mean BMD, trabecular BMD (Tb.BMD), trabecular bone volume ration (BV/TV), trabecular number (Tb.N) and maximum load in the femoral neck of ovariectomized rats. Metaphyseal Tb.BMD and BV/TV were also significantly decreased in the WBVV-treated ovariectomized group than non-treated controls [214].

Few studies [215] have succeeded in restoring BMD or trabecular parameters after the hindlimb unloading period (eight weeks). This last study showed that vibrational loading with one day of rest was substantially effective in improving the architecture and apparent- and tissue-level mechanical properties of the rat distal femoral metaphysis. Moreover, a recent study showed that low-magnitude, high-frequency vibration (LMHFV) with rest days (particularly seven rest days) was considerably effective in improving the morphological and mechanical properties of rat proximal femur. This study may provide an improved understanding of the site-specific responses of bone tissue to LMHFV with rest days for a substantially effective therapy of a targeted bone site in hindlimb rats [216].

#### 4.5.3. Specific Changes in Osteoblast/Osteocyte Lineage Biology

##### Healthy Rats

Very few publications have explored the effect of WBVV on the cell viability and the enzymatic activity (differentiation potentiality) of the osteoblastic/osteocytic lineage [174,175].

Tezval et al. submitted sixty, three-month old female Sprague Dawley rats to sham operation. After three months, this experimental group was divided into two subgroups. In one of the subgroups, rats were treated with WBVV at 90 Hz (3.9 g) for 35 days; the second subgroup remained untreated. During the protocol, new cortical bone apposition was measured after alizarin red and tetracycline subcutaneous injections. Their apposition band widths of fluorochrome labelling were analyzed quantitatively for cortical surface in cross sections acquired in the sub-trochanteric region of the femurs 15 mm distal from the femoral head. The vibration did not show any significant changes on the endosteal surface [175].

WBVV seemed to have an influence on the periosteal side of the cortex (absolute apposition bandwidth (mcm): Sham + WBVV: 14.27 ± 3.77 vs. Sham: 13.15 ± 2.21 after tetracycline injection between day 24 and day 35), but the results were not statistically significant (*p* > 0.05).

##### Osteopenic Models

Falcai et al. compared different protocols of exercise applied on a hindlimb-suspended model in female Wistar rats (220 ± 10 g at the beginning). Their objective was to compare the effects of swimming, jumping and vibration therapies on the prevention of bone loss. They evaluated histomorphometric parameters of bone formation and serum levels of bone markers. Their results have shown that for the specific vibration program, the trained rats showed a significant increase in bone formation and serum levels of bone markers compared to the hindlimb suspension condition [174].

Tezval et al. submitted sixty, three-month old female Sprague Dawley rats to sham operation or ovariectomy. After three months, each experimental group was divided into two subgroups. In one of the subgroups, rats were treated with WBVV at 90 Hz (3.9 g) for 35 days; the second subgroup remained untreated. During the protocol, new cortical bone apposition was measured after alizarin red and tetracycline subcutaneous injections. Their apposition band widths of fluorochrome labelling were analyzed quantitatively for cortical surface as previously described.

They showed that WBVV seemed to have an influence on the periosteal side of the cortex in the OVX group (absolute apposition bandwidth (mcm): OVX + WBVV: 14.84 ± 3.79 vs. OVX: 13.51 ± 5.28 after tetracycline injection between day 24 and day 35), but the results were not statistically significant (*p* > 0.05). The vibration did not demonstrate any significant changes on the endosteal surface [175].

#### 4.5.4. Specific Changes in Osteoclast Biology

One publication describes controversial effects of the WBVV model on the osteoclast activity [174].

##### Osteopenic Models

Indeed, when Falcai et al. compared the unloading interrupted by vibration therapy to the hindlimb unloading suspension condition alone in female rats, they observed that the osteoclast number, the eroded surface and the osteoclastic surface were not statistically different between unloading and unloading interrupted by the vibration condition, even if the values seemed to be lower in this case [174].

## 5. Final General Discussion

The huge variety of protocols used (time, intensity, speed, duration, inclination) does not help to determine which protocol is the most osteogenic. We can suggest that low-intensity protocols related to slow speed, short duration or short frequency of running sessions may not be sufficient to induce statistically significant effects on BMD.

Recently, a study compared two types of running protocol (i.e., continuous vs. intermittent). Using a 30% shorter training period, the intermittent running group (IT) showed more than 20% higher whole-body and femur BMD gains compared to the continuous running group (CT), suggesting that moderate IT was able to produce faster bone adaptations than moderate CT [70].

Some authors have hypothesized that bone responsiveness to exercise should be bone site-specific [48,97,115]. Tibia and femur are considered to be the most responsive bones, but this result can be biased as they are also the most frequently studied. Chang et al. chose to report lumbar BMD instead of distal femur or proximal tibia. They observed a higher BMD variation in femur than in spine and hypothesized that this might be due to a smaller dimension of femoral bone (diameter) [116]. On the other hand, Iwamoto et al. demonstrated that there was no significant effect on lumbar bone mass after moderate continuous running in OVX rats [136]. We can also highlight that it is important to consider that numerous protocols could have different efficacy on whole skeletons. In addition, some other studies have measured BMD by quantitative computed tomography. This technique provides a volumetric (3-dimensional) BMD that has been shown to be a good indicator of bone quality and to correlate well with mechanical testing. Volumetric BMD differs from the areal (2-dimensional) BMD that is obtained using DXA [50,51,52,53,54].

Sometimes, no effect on BMD can be observed, but an increase of biomechanical properties can be seen. As an example, in Hamann et al., even though no difference in Ct BMD was found, Young’s modulus was significantly reduced in downhill running [48], while Young’s modulus is generally linked with cortical BMD. This phenomenon could be associated with changes in microarchitecture, such as collagen fiber orientation. Water content of the bones might also have an influence on bone strength.

Sensitivity to exercise is not only bone-specific but also gender-specific, showing that male rodents’ responsiveness to moderate running exercise is more pronounced than for females [143].

Endurance protocols involve either free activity (wheel running) or compulsory activities (treadmill running or swimming). It is difficult to explain the differences by only the exercise modalities (distance covered). One possibility could be attributed to the effects of the neuroendocrine system and probably to the stress effect of forced exercise.

“Stressors” have been defined as the harmful stimuli that increase the Adrenocorticotropic hormone (ACTH) secretion with consequent glucocorticoid synthesis [217].

If animals are confronted with the stronger psychological and physical stimulus of forced swimming or running than the exercised animals, compared with the controls, they respond with higher glucocorticoid levels [218,219].

The serum ACTH hormone is secreted by the anterior pituitary and is directly responsible for the corticosterone hormone secretion by adrenal glands. The animals submitted to acute running or swimming exercise had significant increases on the serum corticosterone concentrations with small ACTH variations, suggesting high sensibility of the corticosterone synthesis to small serum ACTH modifications.

It has been shown that ACTH has an effect on collagen synthesis in the bone. Moreover, ACTH dose-dependently stimulated osteoblastic cell proliferation and is capable of significantly increasing collagen synthesis in osteoblasts [220]. Moreover, the balance of bone formation and resorption from chronic stress-induced sympathetic activity may shift to favor bone resorption. This is seen with chronic stimulation of b-AR with low-dose agonist treatment in mice, which induces bone loss mainly via enhanced bone resorption, suggesting the control of each cell type by the SNS is temporal [221]. This contrasts with sympathetic nerves, whose contribution to the response to mechanical load seems minimal [222].

High-impact exercise (i.e., jumps) seems to increase bone formation more than aerobic exercise, such as running, in both humans and animals [183,223].

Jump exercise does not require a lot of repetitions to generate significant anabolic effects [181]. Longer intervals (30 s) between individual loading seems to be more effective for anabolic effects than a shorter interval (3 s) and two separated bouts (2 × 10 repetitions) are not more effective than a single bout (1 × 20 repetitions) daily [182]. The mechano-sensors lost sensitivity after several repetitions of constant loading since little additive effects were observed from 10 to 40 repetitions [182]. The mechano-sensor sensitivity is recovered after a low-loading interval [181,182]). Two types of impact training were achieved based on active jumps or passive drops.

Osteocytes are known to be the key mechano-sensors [22] of the bone remodeling. Among the different exercise protocols, literature has shown that treadmill running procedures mainly induced osteogenic effects on the viability of the osteocyte lineage in male, female and ovariectomized rats. About the other modes, we have noticed that (i) running in voluntary wheels has contributed to a negative effect on bone metabolism in old male experimental models, (ii) whole-body vertical vibration was not an osteogenic exercise in female and ovariectomized rats, (iii) swimming provided controversial results in female models.

Moreover, among positive bone anabolic effects, moderate aerobic physical exercises such as wheel and treadmill running, VWBV (under specific conditions) and swimming can contribute to an anti-inflammatory action. Indeed, among molecular mechanisms linking loading and bone, exercise-induced myokines could suppress inflammation, a process which is often raised as a consequence of aging, menopause, dysmetabolism and physical inactivity. In addition to the direct effect of the mechanical loading, physical activity might physiologically modulate bone remodeling through the production of myokines [224,225].

About the osteopenic context, physical exercise may prevent age-associated bone loss through osteocyte apoptosis and mitochondrial function modulation. Endurance and resistance exercise have distinct effects on osteocyte viability [226].

Among molecules involved in transduction signaling pathways, bone morphogenetic proteins (BMPs) family and heat shock proteins (HSPs) play a role in osteocytes biology after mechanical loadings. Indeed, BMPs secreted by osteocytes in response to mechanical loading inhibit apoptosis and promote osteocyte survival [227]. Besides, heat shock proteins that can be induced by mechanical stress could contribute to modulate osteocyte viability in both physiological and pathological conditions, suggesting important implications of specific physical exercises [228].

About osteoclast parameters, running in a voluntary wheel for old males, treadmill running programs at high intensity in OVX rats (speed: 30 m/min), and swimming programs in OVX + CAS rats have detrimental consequences.

In conclusion, bone loss and fragility are mediated by sedentary behavior and the aging of the population. In contrast to pharmacological treatments, exercise can provide a worldwide cheap treatment. Based on this review made in rats including running (forced treadmill or voluntary wheel), swimming, resistance exercise (climb or jump or drop) and whole-body vertical vibrations, we could conclude that physical exercise is not always beneficial for bone and has to be well selected amongst the numerous protocols, duration, intensity and loading. Additional studies are needed to characterize and model the best exercise protocol to improve bone quality.

## Supplementary Materials

The following are available online at https://www.mdpi.com/2075-1729/10/10/217/s1, Table S1: Effects of physical exercise for treadmill, wheel activities, swimming, resistance exercise and vibrating platform on bone parameters in healthy (male and female) and osteopenic rats.

## Abbreviations

ACTH	Adrenocorticotropic hormone
a.ES	active erosion surface
BMD	bone mineral density
BMPs	bone morphogenetic proteins
BS	bone surface (BS)
BV/TV	bone volume/tissue volume
CAS	chronic aversive stimuli
DXA	dual-energy X-ray Absorptiometry
ES	erosion surface
GRF	ground reaction forces
HCRs	high-capacity runners
HSPs	heat shock proteins
It	intermittent running
LCRs	low-capacity runners
LMHFV	low-magnitude, high-frequency vibration
MAR	metaphyseal mineral apposition rate
Oc.S	osteoclast surface
OPG	osteoprotegerin
OVX	ovariectomized
RANKL	receptor activator of nuclear factor kappa-B ligand
SRT	simulated resistance training
TRAP	tartrate-resistant acid phosphatase
CTx	terminal collagen cross-links
Tb.N	trabecular number
Ct.Th	cortical thickness
Tb.BMD	trabecular BMD
Tb.Sp	trabecular spacing
Tb.Th	trabecular thickness
WBVV	whole-body vertical vibration
