# Supplementary material for: Does Physical Exercise Always Improve Bone Quality in Rats?
Source: Life (Basel). 2020 Sep 23;10(10):217. doi: 10.3390/life10100217 (PMC7598192; doi:10.3390/life10100217)
Supplement: Supplementary file 1 [file life-10-00217-s001.pdf]

life

MDPI

Supplementary Materials

Table S1. Effects of physical exercise for treadmill, wheel activities, swimming, resistance exercise and vibrating platform on bone parameters in healthy (male and female) and osteopenic rats.

Exercise Modalities	Sex Or Bone Status	BMD	Micro-Architecture	Mechanical Properties	Osteoblast / Osteocyte Lineage	Osteoclast Activity
		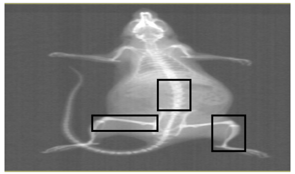	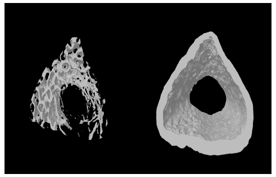	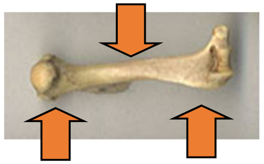	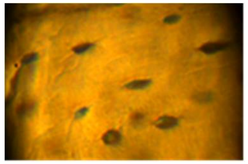	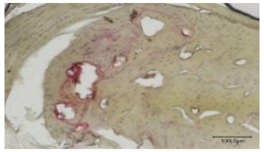
**Treadmill running and walking**						
**Positive effects**						
	**Healthy males**	(Boudenot et al., 2015; Chen et al., 2004; Davicco et al., 1999; Hagihara et al., 2005; Horcajada et al., 1997; Järvinen et al., 2003; Joo et al., 2003; Kannus et al., 1994, 1996; Leppänen et al., 2008; Pajamäki et al., 2003a)	(Huang et al., 2002; Joo et al., 2003; Vicente et al., 2013; Yao et al., 2004)	(Chen et al., 2004; Horcajada-Molteni et al., 1999b; Huang et al., 2008; Järvinen et al., 2003; Joo et al., 2003; Pajamäki et al., 2003b; Takeda et al., 2012)	(Boudenot et al., 2014; David et al., 2007; Iijima et al., 2015; Li et al., 1991; Vicente et al., 2013)	(Bourrin et al., 1995a, 1995b; Swift et al., 2012a; Vicente et al., 2013)
	**Healthy females**	(Hagihara et al., 2009; Hamann et al., 2012a; Iwamoto et al., 2004; Leppänen et al., 2008; Markiewicz-Górka et al., 2019; Omi et al., 1998; van der Wiel et al., 1995; Yeh et al., 1993b, 1993c, 1993a)	(Fang et al., 2019; Iwamoto et al., 1999; Leppänen et al., 2008; Markiewicz-Górka et al., 2019; Swift et al., 2012b; Vicente et al., 2013; Yeh et al., 1993d)	(Fang et al., 2019; Järvinen et al., 1998; Leppänen et al., 2008; Peng et al., 1994)	(Fang et al., 2019)	(Fang et al., 2019; Vicente et al., 2013)
	**Osteopenic**	(Nicolas Bonnet et al., 2007; Chang et al., 2010a; Chen et al., 2011a; Fuchs et al.,	(N. Bonnet et al., 2007; Nicolas Bonnet et al., 2007; Bourrin et	(N. Bonnet et al., 2007; Nicolas Bonnet et al., 2007; Bu et al., 2012;	Barengolts et al. 1994; N. Bonnet et al. 2007; Liu et al. 2012; Ocarino et al. 2007;	(N. Bonnet et al., 2007; Yeh et al., 1993d)

		2007a; Iwamoto et al., 1998; Kannus et al., 1994; Lespessailles et al., 2009; Nakajima et al., 2001; Tamaki et al., 1998a; Yeh et al., 1993d; Yuasa et al., 2020)	al., 1995a; Chen et al., 2011b; Fuchs et al., 2007a; Li et al., 2014a; Ocarino et al., 2010)	Fuchs et al., 2007b; Järvinen et al., 2003; Lespessailles et al., 2009; Peng et al., 1997; Simões et al., 2008a)	Toumi et al. 2015; (Tamaki et al. 1998a; Yeh, Liu, et Aloia 1993)	
**No effect**						
	**Healthy males**	(Boudenot et al., 2012; Horcajada-Molteni et al., 1999a, 1999b; Huang et al., 2002; Iwamoto et al., 2004; Maurel et al., 2013; Notomi et al., 2000; Sipos et al., 2008a; Thomsen et al., 2008; Yao et al., 2004; Yingling et al., 2001)	(Leppänen et al., 2008; Maurel et al., 2011; Notomi et al., 2000; Reed et al., 2002)	(Notomi et al., 2000; Pajamäki et al., 2003c)	∅	∅
	**Healthy females**	(Chang et al., 2010b; Mathey et al., 2002; Oxlund et al., 1998; Tromp et al., 2006; Warner et al., 2006; Yingling et al., 2001)	(Hamann et al., 2012b; Iwamoto et al., 1999; Tromp et al., 2006)	(Fang et al., 2019; Oxlund et al., 1998)	(Swift et al., 2012a; Vicente et al., 2013)	∅
	**Osteopenic**	∅	∅	(Nordsletten et al., 1994; Shimano and Volpon, 2009; Simões et al., 2008b)	∅	∅
**Detrimental effects**						
	**Healthy males**	(Bourrin et al., 1994)	(Bourrin et al., 1994; Maurel et al., 2013; Sipos et al., 2008a)	∅	(Bourrin et al., 1994; Horcajada-Molteni et al., 1999a)	∅
	**Healthy females**	(Rico et al., 1999a, 1999b; Seco et al., 1998; Swift et al., 2012a)	∅	∅	∅	∅
	**Osteopenic**					
**Wheel running**						
**Positive effects**						

	**Healthy males**	(Holy and Zérath, 2000; Newhall et al., 1991; Shiga et al., 2003a)	(Bégot et al., 2011; Thomsen et al., 2008)	(Shiga et al., 2003b)	∅	∅
	**Healthy females**	(Banu et al., 1999)	(Banu et al., 1999; Saino et al., 2003)	(Banu et al., 1999)	(Fonseca et al., 2011a)	∅
	**Osteopenic**	(Li et al., 2014b; Omi et al., 1994)	(Li et al., 2014a)	∅	(Fonseca et al., 2011b; Goulet et al., 2011; Li et al., 2014b)	(Lertsinthai et al., 2015)
**No effect**						
	**Healthy males**	(Sipos et al., 2008b)	(Hydock et al., 2008; Sipos et al., 2008a)	∅	∅	∅
	**Healthy females**	∅	(Hydock et al., 2008)	∅	∅	∅
	**Osteopenic**	(Bodnar et al., 2012a; Hertrampf et al., 2006)	(Bodnar et al., 2012b; Hertrampf et al., 2006)	∅	(Li et al., 2014a)	∅
**Detrimental effects**						
	**Healthy males**	∅	∅	∅	(Sipos et al., 2008b)	(Sipos et al., 2008b)
	**Healthy females**	∅	∅	∅	∅	∅
	**Osteopenic**	∅	∅	∅	∅	∅
**Swimming**						
**Positive effects**						
	**Healthy males**	(Kang et al., 2019)	(Kang et al., 2019)	(Huang et al., 2003)	∅	∅
	**Healthy females**	(Snyder et al., 1992; Swissa-Sivan et al., 1992, 1990)	∅	(Simkin et al., 1989)	∅	∅
	**Osteopenic**	(Karatosun et al., 2006; Volpon et al., 2015)	(Volpon et al., 2015)	(Hart et al., 2001; Volpon et al., 2015)	∅	∅
**No effect**						
	**Healthy males**	(Huang et al., 2010, 2003)	∅	(T. H. Huang et al. 2003)	∅	∅
	**Healthy females**	∅	(Simkin et al., 1989)	∅	(Falcai et al., 2015)	∅
	**Osteopenic**	∅	∅	∅	∅	∅
**Detrimental effects**						

	**Healthy males**	∅	(Huang et al., 2010)	∅	∅	∅
	**Healthy females**	∅	(Bourrin et al., 1992)	∅	(Bourrin et al., 1992)	∅
	**Osteopenic**	∅	∅	∅	∅	∅
**Resistance training**						
**Positive effects**						
	**Healthy males**	(Cavalié et al., 2003; Notomi et al., 2001, 2000; Smith et al., 2008; Swift et al., 2010)	(Ju et al., 2014; Notomi et al., 2001; Swift et al., 2010)	(Notomi et al., 2001)	∅	(Notomi et al., 2001)
	**Healthy females**	(Aikawa et al., 2019; Honda et al., 2008; Rosa et al., 2010; Umemura et al., 1995; Welch et al., 2008, 2004)	∅	(Aikawa et al., 2019; Honda et al., 2008; Umemura et al., 2002, 1997; Welch et al., 2008, 2004)	∅	∅
	**Osteopenic**	(Falcai et al., 2015; Honda et al., 2001, 2003; Ju et al., 2008, 2012, 2013, 2014; Notomi et al., 2001; Okubo et al., 2017; Swift et al., 2011a; Umemura et al., 1995, 2008b; Yanagihara et al., 2016)	(Notomi et al., 2014; Okubo et al., 2017; Swift et al., 2011a)	(Honda et al., 2003, 2001; Notomi et al., 2014)	(Falcai et al., 2015; Swift et al., 2011b)	(Falcai et al., 2015; Macias et al., 2011)
**No effect**						
	**Healthy males**	(Notomi et al., 2001; Smith et al., 2008)	∅	∅	∅	∅
	**Healthy females**	(Umemura et al., 2008a)	(Aikawa et al., 2019)	(Lin et al., 2013; Nagasawa et al., 2008)	∅	∅
	**Osteopenic**	∅	∅	∅	∅	∅
**Detrimental effects**						
	**Healthy males**	∅	∅	∅	∅	∅
	**Healthy females**	∅	∅	∅	∅	∅
	**Osteopenic**	∅	∅	∅	∅	∅

**Vibrations**						
**Positive effects**		**BMD**	**Micro-architecture**	**Mechanical properties**	**Osteoblast / osteocyte lineage**	**Osteoclast**
	**Healthy males**	(Yang et al., 2009a)	(Gong et al., 2019; Ogawa et al., 2014; Pasqualini et al., 2013)	(Gong et al., 2019; Yang et al., 2009a)	∅	∅
	**Healthy females**	(Maddalozzo et al., 2008)	∅	∅	∅	∅
	**Osteopenic**	(Liang et al., 2014)	(Hatori et al., 2015)	∅	(Falcai et al., 2015; Tezval et al., 2011)	∅
**No effect**		**BMD**	**Micro-architecture**	**Mechanical properties**	**Osteoblast / osteocyte lineage**	**Osteoclast**
	**Healthy males**	(Nowak et al., 2014; Yang et al., 2009b)	(Pasqualini et al., 2013)	(Yang et al., 2009b)	∅	∅
	**Healthy females**	(Maddalozzo et al., 2008)	∅	∅	(Tezval et al., 2011)	∅
	**Osteopenic**	(Camargos et al., 2015a; Hoffmann et al., 2016, 2017; Komrakova et al., 2016a)	(Camargos et al., 2015a; Hatori et al., 2015; Hoffmann et al., 2016, 2017; Komrakova et al., 2016a)	(Camargos et al., 2015b; Hoffmann et al., 2017, 2016; Komrakova et al., 2016b)	(Tezval et al., 2011)	∅
**Detrimental effects**						
	**Healthy males**	∅	∅	∅	∅	∅
	**Healthy females**	∅	∅	∅	∅	∅
	**Osteopenic**	(Xie et al., 2016)	(Xie et al., 2016)	(Xie et al., 2016)	∅	(Falcai et al., 2015)

Positive effects: means that bone parameters were increased compare to control, **No effect:** means that bone parameters did not differ from control, **Detrimental effects:** means that bone parameters were decreased compare to control, ∅: Means that no results were found in the reported literature.
